# Short-Term Psychodynamic Psychotherapy in Patients with “Male Depression” Syndrome, Hopelessness, and Suicide Risk: A Pilot Study

**DOI:** 10.1155/2013/408983

**Published:** 2013-01-14

**Authors:** Gloria Angeletti, Maurizio Pompili, Marco Innamorati, Chiara Santucci, Valeria Savoja, Mark Goldblatt, Paolo Girardi

**Affiliations:** ^1^Department of Neurosciences, Mental Health and Sensory Functions and Department of Psychiatry, Sant'Andrea Hospital, Sapienza University of Rome, 00189 Rome, Italy; ^2^Department of Neurosciences, Mental Health and Sensory Functions and Department of Psychiatry, Suicide Prevention Center, Sant'Andrea Hospital, Sapienza University of Rome, 1035 Via di Grottarossa, 00189 Rome, Italy; ^3^McLean Hospital, Belmont, MA, USA; ^4^Department of Psychiatry, Harvard Medical School, Cambridge, MA 02138, USA; ^5^Villa Rosa Medical Research Centre, Viterbo, Italy

## Abstract

*Objectives and Methods*. This was an observational study of the efficacy of short-term psychodynamic psychotherapy (STPP) in a sample of 35 (30 women and 5 men) patients with moderate-to-severe “male depression” (Gotland Scale for Male Depression (GSMD) ≥ 13) comorbid with unipolar mood disorder (dysthymia and major depression) or anxiety disorder. Outcome measures were GSMD and BHS (Beck Hopelessness Scale) score changes from baseline. *Results*. Patients had a strong response to STPP on the GSMD (estimated mean score change (± SE) = −9.08 ± 2.74; *P* < 0.01; partial eta squared = 0.50), but not on the BHS (estimated mean score change (± SE) = −0.92 ± 1.55; *P* = 0.57; partial eta squared   = 0.03). BHS score changes were significantly associated with GSMD score changes (Pearson's *r* = 0.56; *P* < 0.001), even when controlling for the severity of hopelessness at the baseline (partial *r* = 0.62; *P* < 0.001). *Conclusions*. STPP proved to be effective in patients suffering from “male depression” although hopelessness was only marginally reduced by this treatment which points to the need to better understand how STPP can be involved in the reduction of suicide risk.

## 1. Introduction

The term “depression” encompasses a wide range of conditions that may occur along a continuum, ranging from milder forms of discomfort to more severe and persistent form, as in the case of major depression. Depression is the leading cause of disability and the 4th leading contributor to the global burden of disease [1, 2] and by the year 2020, it is projected to become the 2nd leading contributor to the global burden of disease in all ages and both sexes [1].

Major depression is the most frequent mental illness in the world [3–6]. For example, in the US, the Epidemiological Catchment Area (ECA) Study indicated a one-month prevalence between 1.7% and 3.4% [7], and more recently, the National Comorbidity Survey Replication (NCS-R) estimated a 12-month prevalence of 6.6% [8]. Nevertheless, prevalence of moderate- to- severe depressive symptoms could be much higher [9–11].

In 2010, the British National Institute for Health and Clinical Excellence (NICE) commissioned the development of an updated version of the guideline on the treatment and management of depression in adults [12]. The NICE guideline pointed out that people who suffer from depression usually prefer psychological treatments to medication [13] and value outcomes beyond symptom reduction [14]. The NICE guideline indicated that it was not possible to demonstrate a consistent picture of any clinically important benefit for short-term psychodynamic psychotherapy (STPP) in depression. While cognitive-behavioral therapy and interpersonal therapy continue to have the most evidence for efficacy [15], however, some randomized trials and meta-analyses indicated that STPP could be effective in reducing symptoms and in improving functional ability of patients with mild or moderate depression [16–20].

Based on the experiences of the Gotland Study, Wålinder and Rutz [21] identified a male depressive syndrome including low stress tolerance, acting-out behavior, poor impulse control, substance abuse and family history of depression, alcoholism, and suicidality. The authors also devised an instrument to measure such syndrome, the Gotland Scale for Male Depression [22]. Despite its name, male depression is common in women; for example, female students showed a greater risk of male depression than their male counterparts in one study [23]. Male depression was found to be equally severe in men and women who had made a nonviolent suicide attempt [24]. Both men and women with substance abuse have a higher probability of having male depression and higher suicide risk than those without substance abuse [25].

To date, no psychological therapies have been assessed for their efficacy in the treatment of male depression. Thus, the aim of this study was to assess the efficacy of STPP in reducing “male depression” symptoms comorbid with mood disorders or anxiety disorders in an outpatient setting. Our hypothesis is that STPP can be effective in reducing male depression. Furthermore, we hypothesize that the reduction in male depression will be significantly associated with a reduction of hopelessness, a proxy of suicide risk.

## 2. Materials and Methods

### 2.1. Participants

 This was an observational study of the efficacy of STPP in a sample of 35 consecutive (30 women and 5 men) outpatients with moderate to severe “male depression” (*Gotland Scale for Male Depression* (GSMD) ≥ 13) comorbid with an unipolar mood disorder (dysthymia and major depression) or an anxiety disorder. Patients were admitted between January and June 2009 at the Department of Psychiatry of the Sant'Andrea Hospital, Rome, Italy. Inclusion criteria were the presence of moderate- to- severe “male depression” syndrome as assessed by the GSMD, ages between 18 and 64 years old, and a diagnosis of a mood disorder or anxiety disorder. Exclusion criteria were the presence of lifetime diagnosis of delirium, dementia, amnesic or other cognitive disorders, schizophrenia or other psychotic disorders, anorexia nervosa and bulimia nervosa, comorbid cluster B personality disorder, current presence of severe suicide intent, a score on the *Hamilton Depression Rating Scale* [26] of 28 or higher, and the inability to complete the assessment for illiteracy or the denial of informed consent. 

As concomitant psychotropic medication, only benzodiazepines at a maximum of 3 mg lorazepam equivalent per day were allowed.

All the patients participated voluntarily in the study and gave their informed consent. The study was approved by the local IRB.

### 2.2. Measures

At intake the participants were assessed for psychopathology by expert clinical psychologists through a clinical interview based on the DSM-IV-TR [27]. The patients were also administered the GSMD and the Beck Hopelessness Scale (BHS: [28]). 

The GSMD [21, 22, 29] is a screening instrument for male depression, consisting of 13 items which are rated on a 4-point Likert scale from 0 (not present) to 3 (present to a high degree). A score of 12 or lower indicates no depression, scores in the 13–26 range indicate moderate depression, and scores >26 indicate severe depression [22]. The GMDS has good validity [30–32].

The BHS is a 20-item scale for measuring the cognitive component of the syndrome of depression. This scale assesses three major aspects of hopelessness: feelings about the future, loss of motivation, and expectations. Responding to the 20 true or false items on the Beck Hopelessness Scale, individuals have to either endorse a pessimistic statement or deny an optimistic statement. Research consistently supports a positive relationship between BHS scores and measures of depression, suicidal intent, and current suicidal ideation. In addition, Beck et al. [33] carried out a prospective study of 1,958 outpatients and found that BHS scores were related significantly to eventual completed suicide. A cutoff score of 9 or above identified 16 (94%) of the 17 patients who eventually committed suicide. The high-risk group identified by this cutoff score was 11 times more likely to commit suicide than the rest of the outpatients. The BHS may, therefore, be used as an indicator of suicide potential. An Italian version of the BHS has validated by the authors of the present study [34, 35]. 

### 2.3. Interventions

Our intervention was derived from Malan's focused short-term technique [36, 37]. 

The treatment was administered in individual 45-minute sessions per week for no longer than 40 sessions. STPP explores those aspects of self not fully known, especially as they are manifested and potentially influenced in the therapy relationship [38]. It requires the psychological work to be organized around a focus (i.e., a specific, strategic conflictual area to reach an understanding of the psychopathological picture manifesting as a crisis), and that the therapist takes an active role working through the central conflictual area in the psychic life of the patient.

The psychotherapists were psychologists or psychiatrists certified as psychotherapists from the Italian Board with a previous training in STPP. Group supervision sessions were carried out weekly allowing psychotherapists to discuss their cases with senior psychotherapists/supervisor.

### 2.4. Analysis

The primary outcome measure was the mean GSMD score changes from baseline. The secondary outcome was mean score changes from baseline on the BHS. Response to treatment was assessed with a general linear model for repeated measures. Differences between baseline and followup are calculated on the estimated marginal means (*M*_*e*_). Partial eta squared (*η*_*p*_^2^) are reported as measures of effect size. Associations between response on the GSMD and on the BHS were assessed through Pearson's *r* and partial indices of correlations. All the analyses were performed with the statistical package for social sciences SPSS for Windows 19.0.

## 3. Results

The mean age of the patients was 40.0 years (SD = 12.6; Min./Max.: 20/62), 40.3 ± 19.6 years for men and 40.0 ± 12.1 years for women. Sixty percent of the patients had a diagnosis of anxiety disorders (mostly, generalized anxiety, panic attack disorder, and anxiety disorder not otherwise specified), 26% were diagnosed with dysthymia, and 14% were diagnosed with a major depressive disorder.

Psychotherapy sessions were carried out for a mean of 7.2 months (SD = 2.0; range, 5–10) (see Table 1). At baseline, mean GSMD scores were 23.6 (SD = 6.1; range, 14–37). Sixty-three percent of patients scored 9 or more on the BHS at baseline (10.5 ± 4.9).

Patients had a significant strong response to the psychodynamic psychotherapy on the GSMD (*M*_*e*_ ± SE = −9.08 ± 2.74; *P* < 0.01; *η*_*p*_^2^ = 0.50), but not on the BHS (*M*_*e*_ ± SE = −0.92 ± 1.55; *P* = 0.57; *η*_*p*_^2^ = 0.03). Mean changes on outcome measures were neither associated with the length of the treatments (Pearson's *r* from −0.06 to 0.16), nor with the baseline severity of male depression (Pearson's *r* between 0.05 and 0.07), or hopelessness (Pearson's *r* = 0.05). Only mean BHS score change was moderately associated with baseline severity of hopelessness (*r* = 0.50; *P* < 0.01).

Furthermore, mean GSMD score change was significantly associated with BHS score change (Pearson's *r* = 0.56; *P* < 0.001), even when controlling for the severity of hopelessness at the baseline (partial *r* = 0.62; *P* < 0.001).

## 4. Discussion

The results of this pilot study of short-term psychodynamic psychotherapy for “male depression” are mixed. We found that self-ratings of depressive symptoms were significantly improved, suggesting that this therapy is effective for depressive symptoms in these patients.

Self-ratings associated with increased suicide risk were not significantly affected over the course of treatment. Even those patients who reported a significant response on the MINI-based suicidal risk after treatment showed no improvement on the BHS. This result is consistent with other studies, such as that of Linehan et al. [39], which showed a reduction in the risk of suicide after a psychotherapeutic intervention, but no significant changes between baseline and posttreatment in BHS scores. However, cognitive behavioural therapy proved to be superior in the reduction of hopelessness (a proxy of suicide risk) compared to other therapeutic interventions [40].

Although suicide risk has most often been studied in the context of depressive symptoms, it remains unclear what distinguishes those depressions with lethal outcome from those in which the patients do not attack themselves.

It should also be noted that these treatments took place in a public psychiatric setting where their sessions are part of the government supported public health system, and patients paid a small additional fee. While there are differences between sessions taking place in a therapist's office and those in the rooms of an outpatient clinic (due to changes in the setting from time to time, hospital furniture, white coats around, etc.), every effort was made to maintain standard procedures and provide a good test of STPP. Nevertheless, we must admit that discussion over the delivery of such psychotherapy in public environment was often raised nationwide, pointing to caution in the generalization of our results.

Such important considerations may explain deficits in outcomes as compared with modalities in which STPP is currently practiced.

It should also be noted that our original model was derived from Malan's technique; our approach shared principles with Davanloo's method [41, 42]. Although it is generally acknowledged that Malan espoused the methods of Davanloo, seeing in them a radical fulfilment of his own work, recent eminent evidence also support a valid role for Malan's approach. Malan's proposals, both in direct or modified forms, have been thoroughly tested in efficacy studies [43]. Moreover, common principles among short-term psychotherapies can be identified, which points to the fact that Malan and Davanloo's approaches have similarities that are nowadays acknowledged among experts [44].

This study has noteworthy limitations, including moderate sample size and treatment duration, mix of diagnoses, nonrandomized and largely uncontrolled treatment regimens, including use of benzodiazepine—all of which may limit generalization of findings. However, all subjects were selected for having specific features of depression and suicide risk so that the sample was overall homogeneous. Despite the fact that all psychotherapists involved in this study were trained under the principles of the brief psychodynamic psychotherapy [45], treatment bias might have been appeared, even though all the therapists were routinely supervised by senior psychotherapists. Although all psychotherapists took careful notes and reported detailed description of each session, there was no videotape or audiotape. No doubt, this constitutes a limitation, however, concordant with the limited facilities provided by the public service.

## 5. Conclusions

This study of the use of short-term psychodynamic therapy for “male depression” with features of suicidality reveals how difficult it is to clearly address the suicide risk factors in such patients. Although depressive symptoms appear to improve relatively soon after initiating therapy, suicidality lingers for a much longer time. Further research is needed to clarify the positive effects of such therapy and the interaction between depression and suicidality. Further studies are also needed to distinguish what kinds of therapies are useful in rapidly addressing suicide risk.

## Figures and Tables

**Table 1:** Outcomes.

Variables		Estimated differences (*M* ± SE)	*P*<	Effect size (partial eta squared)
Men—%	14.3			
Women—%	85.7			
Age—*M* ± SD	40.00 ± 12.57			
Therapy duration—months	7.21 ± 2.03			
Baseline BHS—*M* ± SD	10.53 ± 4.90	−0.92 ± 1.55	0.57	0.03
Follow-up BHS—*M* ± SD	9.03 ± 5.17			
Baseline GMDS—*M* ± SD	23.57 ± 6.06	−9.08 ± 2.74	0.01	0.50
Follow-up GMDS—*M* ± SD	15.54 ± 8.89			

Multivariate test of within subject effect: Wilks' *λ* = 0.49; *F*_2;10_ = 5.11; *P* < 0.05; *η*_*p*_^2^ = 0.51.

GMDS ≤ 13: no depression; GMDS between 13 and 26: moderate depression; GMDS > 26: severe depression. BHS ≤ 3: no hopelessness; BHS between 4 and 8: mild hopelessness; BHS ≥ 9 moderate- to- severe hopelessness.
